# Epidemiological study on *Schistosoma mansoni* infection in Sanja area, Amhara region, Ethiopia

**DOI:** 10.1186/1756-3305-7-15

**Published:** 2014-01-09

**Authors:** Getachew Alebie, Berhanu Erko, Mulugeta Aemero, Beyene Petros

**Affiliations:** 1 Department of Biology, Jigjiga University, P.O. Box 1020 Jigjiga, Ethiopia; 2 Aklilu Lemma Institute of Pathobiology, Addis Ababa University, P. O. Box 1176 Addis Ababa, Ethiopia; 3 Department of Biology, College of Natural and Computational Sciences, University of Gondar, P. O. Box 941 Gondar, Ethiopia; 4 Microbial, Cellular and Molecular Biology Department, College of Natural Sciences, Addis Ababa University, P. O. Box 1176 Addis Ababa, Ethiopia

**Keywords:** *Schistosoma mansoni*, New transmission foci, Epidemiology, Ethiopia

## Abstract

**Background:**

The epidemiology of schistosomiasis is well documented and its geographic distribution has been mapped and there is an ongoing mapping in Ethiopia. Nevertheless, new transmission foci have been discovered in different parts of the country. The objective of this study was to assess the establishment of transmission and determine the prevalence of *Schistosoma mansoni* infection in school children from Sanja Town, northwest Ethiopia.

**Methods:**

A cross-sectional parasitological survey involving 384 school children in two primary schools of Sanja Town was conducted between February and April 2013. Stool specimens were collected and microscopically examined using Kato-Katz and Sodium acetate-acetic acid-formalin (SAF) concentration methods. Malacological survey was also carried out to identify snail intermediate hosts and larval infection rate in the snail. The snails collected were checked for trematode infection by shedding. Observation was also made on water contact habits of the study population.

**Results:**

The prevalence of *Schistosoma mansoni* infection using Kato-Katz method was high among male (79.5%) children in Sanja Primary school while it was high among female (75%) children in Ewket Amba Primary school. The prevalence of *Schistosoma mansoni* infection among Sanja Primary school children in the age groups 5–9 and 10–14 years were 84.6% and 75.2%, respectively while in Ewket Amba Primary school, the prevalence was 66% and 77.9% in the age groups 5–9 and 10–14 years respectively. The prevalence of *schistosome* infection in *Biomphalaria pfeifferi* was 16.9% and 0.027% during February and April, respectively. *S. mansoni* infection was successfully established in laboratory mice and adult worms were harvested after six weeks of laboratory maintenance. Observations made on water contact activities showed swimming, bathing and washing in the river and the stream as the high risk activities for *Schistosoma mansoni* infection.

**Conclusion:**

The study has shown establishment of transmission of schistosomiasis mansoni in Sanja Town. Therefore, appropriate integrated control measures need to be introduced to reduce morbidity in the population and also to control the transmission of schistosomiasis in the study area.

## Background

Schistosomiasis is one of the most widespread of all human parasitic diseases, ranking second only to malaria in terms of its socioeconomic and public health importance in tropical and subtropical areas [1].

In an attempt to measure the global burden of schistosomiasis, various estimates have been made. In 2007, the World Health Organization estimated 235 million cases of schistosomiasis worldwide, with 732 million people at risk of infection in known transmission areas [2] but under-diagnosis, when combined with co-morbid conditions that are competing causes of disease (e.g., hookworm or malaria), is not taken into account [3]. If these WHO values are adjusted for the probable 40–60% of missed diagnoses [4-6], the true number of active schistosome infections in 2007 was more likely between 391 and 587 million people worldwide [3]. According to previous estimates, the disease causes the annual loss of between 1·7 and 4·5 million disability adjusted life years (DALYs) [7].

In Africa the great majority (80-85%) of schistosomiasis is found in sub-Saharan Africa [8], where *S. haematobium*, *S. intercalatum* and *S. mansoni* are endemic. However, the two main causative species of schistosomiasis are *S. haematobium* and *S. mansoni*. In this sub-continent, approximately 393 million people are at risk of infection from *S. mansoni*, of which 54 million are infected. A previous estimate for *S. haematobium* infection showed that about 436 million are at risk, of which 112 million are infected [9].

Numerous factors act to determine the transmission of schistosomiasis, directly or indirectly, by affecting the transmission cycle of the schistosome parasite [10]. Each case of schistosomiasis, transmission is enabled by the interrelated effects of broader environmental, climatic, biological, political, demographic, economic, social and cultural trends [11]. Ecological changes resulting from water resource development and/or population movement are also important determinants of the epidemiology of schistosomiasis [12].

Schistosomiasis is also one of the most important parasitic diseases in Ethiopia. Temperature appears to be the major factor that affects the distribution of both *Schistosoma* species (*S. mansoni* and *S. haematobium*) in Ethiopia. *S. mansoni* is found mainly at altitudes between 1200-1900 m above sea level. *Biomphalaria* are the obligate intermediate hosts of *S. mansoni*. Two species of the genus *Biomphalaria*, *B. sudanica* and *B. pfeifferi*, are known to transmit *S. mansoni* in Ethiopia [13]. Unlike, *B. pfeifferi*, which is known to have a wide geographical distribution, *B. sudanica* has very limited distribution in Ethiopia. Its presence has so far been reported from only three areas in the Rift Valley, Ziway and Abaya Lakes and the interface between Tikur Wuha River and Awassa Lake [14]. It seems, therefore, that *B. pfeifferi* has ubiquitous distribution, while *B. sudanica* is limited in its distribution.

*Bulinus abyssinicus* and *Bu. africanus* are the only bulinid species found naturally transmitting *S. haematobium* in Ethiopia, even though about 10 *Bulinus* species are expected to occur [15]. From the previous studies it is established that *Bu. abyssinicus* is the intermediate host for *S. heamatobium* in Awash valley, whereas *Bu. africanus* transmit the disease in Kurmuk (an area located near to Sudan) [12].

Generally new transmission foci are being discovered in different parts of the country over time. The reasons for the spreading of the disease to new localities seem to be the establishment of water resource development projects such as dams and irrigation and migration of people from endemic areas to previously non-endemic ones [16].

Currently, the government of Ethiopia has given more attention to the development of dams for hydroelectric power such as Gilgel Gibe hydroelectric dam and irrigation based agriculture schemes. These developments inevitably lead to the spread of schistosomiasis unless careful planning is made and timely interventions are put in place in areas where transmission has already been established.

Based on information obtained from health centers in Sanja area, a large number of schistosomiasis cases are diagnosed in young children who were born and brought up in the +area. However, epidemiological studies have not been conducted in the area to find out the e+stablishment of transmission. The present epidemiological study was, therefore, conducted to determine the transmission and magnitude of *S. mansoni* and the prevalence of other intestinal parasites in this area.

## Methods

### Study area

**Figure 1:**
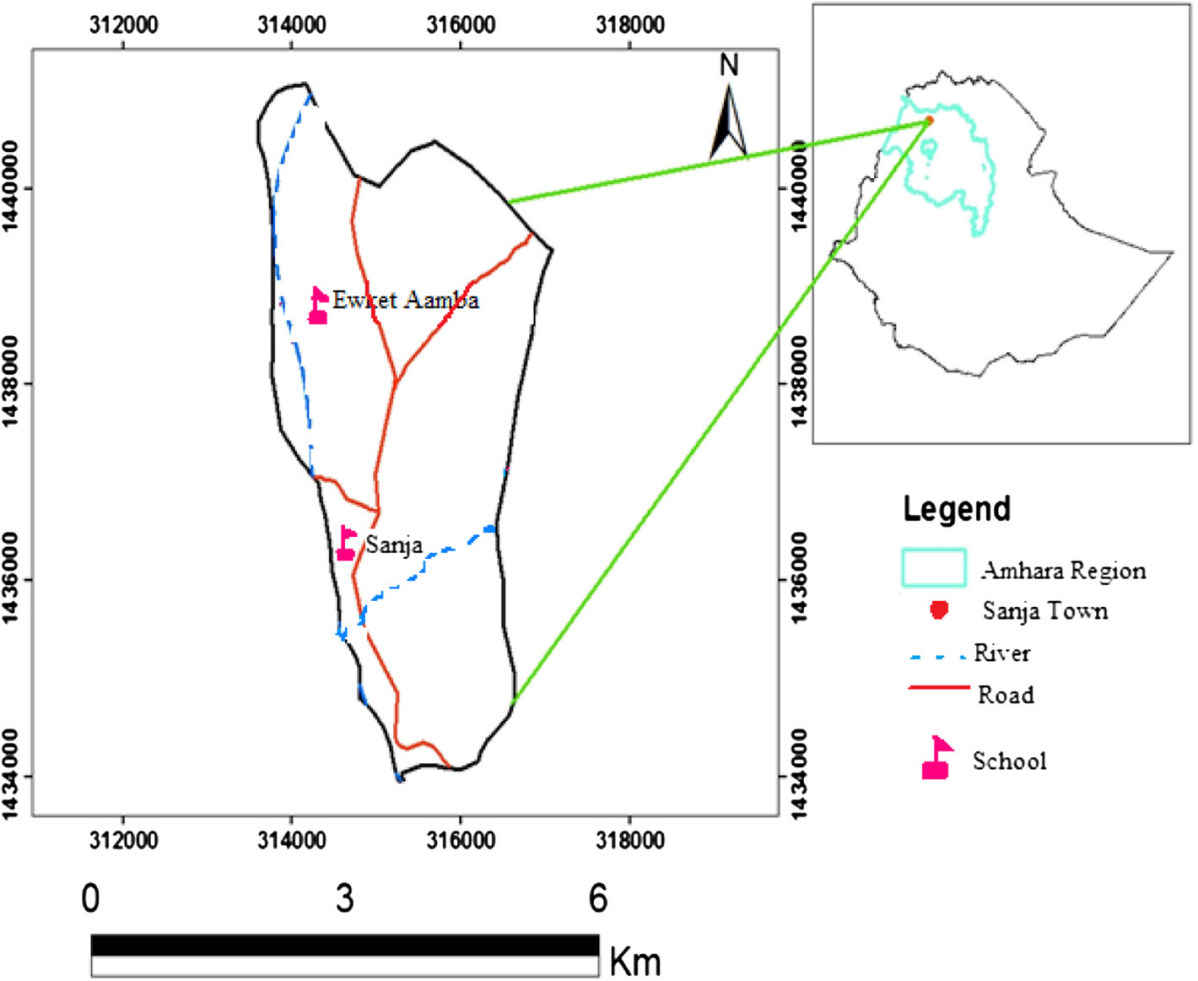
Map of study area.

The study was conducted in Sanja Town, located about 792 km away from Addis Ababa in the northwest of Ethiopia, Amhara region, north Gondar Zone (Figure 1). The area has altitudinal ranges of 1900 to 2200 masl with N 12°59′E 37°18′ coordinates. The two distinct seasons in the area are the wet/raining and dry season. The wet season lasts from April to October, characterized by slight rains in April and May and heavy rains with subsequent flooding of banks of rivers in July and August. The dry season lasts from November to March, characterized by high temperature. The annual rainfall is 300 mm to 750 mm, reaching its peak from July to August. The temperature is warm and ranges from 29°C to 31°C.

The town of Sanja is traversed by one river and one stream, namely, Sanja River and Maho stream which constitute the major sources of *S. mansoni* infection. Sanja River is large and provides most of the services for the residence where as Maho is a small stream, slow flowing and it was stagnant after February. The town had a population of approximately 7255 inhabitants in 2007, of whom 3591 were males and 3664 were females.

Social service institutions in the area include one major health center and six schools (1 kindergarten, 2 primaries, 1 high school, 1 preparatory and 1 vocational school). The health center had beds and provided diagnostic and treatment services for the inhabitants of the town and the surrounding areas. Standing pipes were supplying water for residents in the village although they were inadequate to meet their demands. Sanja River and Maho stream serves as sources of water for laundering, bathing and other domestic and recreational purposes. Most of the town dwellers had no toilet and excreted their feces in open field, particularly in groves and shades of trees near the two water bodies.

### Study design

A cross-sectional parasitological and malacological study was conducted between January and April 2013 in order to determine the endemicity of *Schistosoma mansoni* in Sanja Town and the associated risk factors.

### Study population

The study population consisted of school children age 5–19 years old attending grades 1–8 in two primary schools (Sanja and Ewket Amba). Sample was divided among the selected schools based on the number of students who were attending in both schools (high number of students were attending in Sanja primary school compared with Ewket Amba primary school as it was newly established). Stool samples were collected from school children by simple random sampling. Selection of students who were included in the study was based on the number of sections per grade and the number of students per sections (relatively small number of students per section were selected if the number of sections per grade were high and high number of students per section were selected if the number of sections per grade were small and with high number of students per section).

### Sample size determination

The sample size (n) was determined using the statistical formula [17] n = Z^2^ p (1-p)/d^2^, Where n = sample size, Z is 95% confidence interval (1.96), P is expected prevalence (50%) and d is precision or margin of error (5%). Since there was no schistosomiasis prevalence report for the area, a prevalence of 50% was assumed. Hence, the required sample size was computed to be 384. The study participants were then selected by systematic random sampling using school roll as a sampling frame.

### Stool sample collection and examination

Stool samples were collected from children enrolled in Sanja and Ewket Amba Primary schools, in Sanja Town. The study objectives were explained to the school directors, teachers and children before stool collection. Each participant was given plastic sheet with applicator stick and was instructed to bring sizable stool sample of his/her own. Kato-Katz and Sodium acetate-acetic acid-formalin (SAF) solution concentration methods were used to process the stool specimen for microscopic examination. Stool samples were collected in dry, clean and labeled stool cups and preserved in 10 ml SAF solution and later processed using saline ether concentration [18]. Qualitative microscopic examination was done to determine the prevalence of intestinal parasites. A single Kato-Katz slide was prepared from each fresh stool sample for both quantitative egg count of *S. mansoni* infection and determination of the presence and absence of other intestinal parasitic infections (except hookworm infection). The egg of *S. mansoni* in the Kato slides was counted and the number of eggs was multiplied by 24 to convert into eggs per gram of stool. The intensity of infection was calculated as the geometric mean for infected children and expressed as eggs per gram of feces (epg). Classes of intensity were categorized as light (epg < 100), moderate (epg between 101 and 400) and heavy epg >400) [19].

### Malacological survey

Sanja River and Maho stream were surveyed for *S. mansoni* intermediate host snails. Snails were collected using a scoop and *B. pfeifferi* intermediate host of *S. mansoni*, and other snails such as *Bulinus forskali* and *Lymnaea natalensis* were identified morphologically using shell morphology [20], and then transferred in to plastic buckets containing water and vegetation. Thereafter, snails were transported to Aklilu Lemma Institute of Pathobiology for determination of infection. The snails were then examined for natural trematode infections by the shedding method. Each snail was placed individually in the shedding vials containing aged water and then exposed to electric light for about one hour. The cercariae shed by the snails were identified to the genus level by their tail morphology. During snail collection, observations were also made on physical characteristics of the habitat such as vegetation abundance, turbidity, the nature of the substrate and speed of the water.

### Mice infection with schistosome cercariae

Laboratory bred mice were exposed to the cercariae shed from the snails. This was done by suspending the mice in a way that the tails of the mice was in infected water. The exposed mice were kept for 30 minutes until infection was established. Mice were sacrificed and worms were collected manually from blood vessels around the mesentery after 6 weeks of maintenance. Definite identification of the schistosome parasite was made based on the egg and adult worm morphology.

### Interview with structured questionnaire

Sociodemographic data which include sex, age, religion and other necessary information were gathered. Presence of *S. mansoni* infection risk factors like swimming habit, washing clothes in the river, any river water contact during crossing, and source of water for cooking and drinking were asked from the study populations. Direct observations were also made on these risk factors.

### Data analysis

The data were analyzed using SPSS Version-16 software. Cross tabulation was used to determine the prevalence of *S. mansoni* and other intestinal parasitic infection. Prevalence and intensity of *S. mansoni* infection were reported in percent and mean egg count respectively. The association of *S. mansoni* infection with water contact habits and demographic factors were statistically tested using bivariate regression analysis. The magnitude of association was measured through odds ratio at 95% confidence interval and p-value of <0.05 was considered to be significant.

### Ethical considerations

Ethical clearance to conduct the research was obtained from the Ethical Clearance Committee of the Faculty of Life Sciences, Addis Ababa University. Permission was also obtained from North Gondar Health Bureau, Sanja Health and Education Bureaus to conduct the study. The objectives of the study were explained to the directors, teachers, students and parents of the study participants after which informed consent was obtained from the students and their parents (assent in case of children younger than 15 years old). Those children found positive for *S. mansoni* were treated with a single dose of Praziquantel (40 mg/kg body weight). Children who were found positive for STHs were treated with albendazole (400 mg) [19].

## Results

### Socio-demographic findings

A total of 384 individuals with age range of 5 to 19 years old were included in the study from two primary schools in this study. Out of this, 248 (64.58%) were from Sanja Primary school and the rest 136 (35.42%) were from Ewket Amba Primary school. The numbers of female and male students in Sanja primary school were 126 (50.8%) and 122 (49.2%) respectively whereas the numbers of female and male students in Ewket Amba primary school were 72 (52.9%) and 64 (47.1%) respectively.

### Parasitological investigations

Microscopic examination of stool was done using SAF concentration technique and Kato-Katz methods. Results of Kato-Katz and SAF concentration methods were obtained for 384 school children. Pooling the results of the two diagnostic approaches (positive in either method) increased the prevalence of both *S. mansoni* and other intestinal parasitic infection (Table 1).

**Table 1:** Prevalence of intestinal parasitic infection as determined by Kato-Katz and SAF concentration methods

**Infection by**	**Number (%)**				
	**Kato-Katz method (n = 384)**	**SAF concentration method (n = 384)**	Combined results*		
			**Male(n = 187)**	**Female(n = 197)**	**Both sexes(n = 384)**
*S. mansoni*	293(76.3)	212(55.2)	157(83.9%’)	161(81.7)	318(82.8)
Hookworm	0(0)	42(10.9)	18(9.6)	24(12.1)	42(10.9)
*A .lumbricoides*	3(0.8)	3(0.8)	4(2.1)	2(1.0)	6(1.6)
*T. trichuria*	1(0.3)	0(0)	1(0.5)	0(0)	1(0.3)
*Taenia* species	1(0.3)	3(0.8)	1(0.5)	2(1.0)	3(0.8).
*E. vermicularis*	2(0.5)	1(0.3)	1(0.5)	2(1.0)	3(0.8)
*H. nana*	0(0)	1(0.3)	1(0.5)	0(0)	1(0.3)
At least one intestinal parasites	295(76.8)	219(57)	162(86.6)	165(83.8)	327(85.2)

*Combined result indicates any child that was positive either by Kato-Katz or SAF.

Of the total 384 children examined, 327 (85.2%) were found positive for various intestinal parasites (after pooling the results of the two diagnostic methods (Table 1). The most prevalent parasitic infection was intestinal schistosomiasis (82.8%) due to *S. mansoni*. Out of this prevalence, (48.7%) were positive by both methods while (27.6%) and (6.5%) were positive only by Kato-Katz and SAF concentration methods respectively. Although the prevalence of *S. mansoni* was slightly higher among males (83.9%) than females (81.7%), the difference was not statistically significant (p > 0.05). The number of school children with detectable soil transmitted helminth infection was low. While 42 (10.9%) children had hookworm 6 (1.6%) had *A. lumbricoides*. Both *Taenia* species and *E. vermicularis* had low prevalence (0.8%). Other rare parasites encountered in this study were *T. trichuria* and *H. nana*, each 0.3% (Table 1).

By taking the Kato-Katz method as a gold standard for detecting *S. mansoni* egg the specificity and sensitivity of the SAF concentration method was 72.5% and 63.6% respectively.

**Figure 2:**
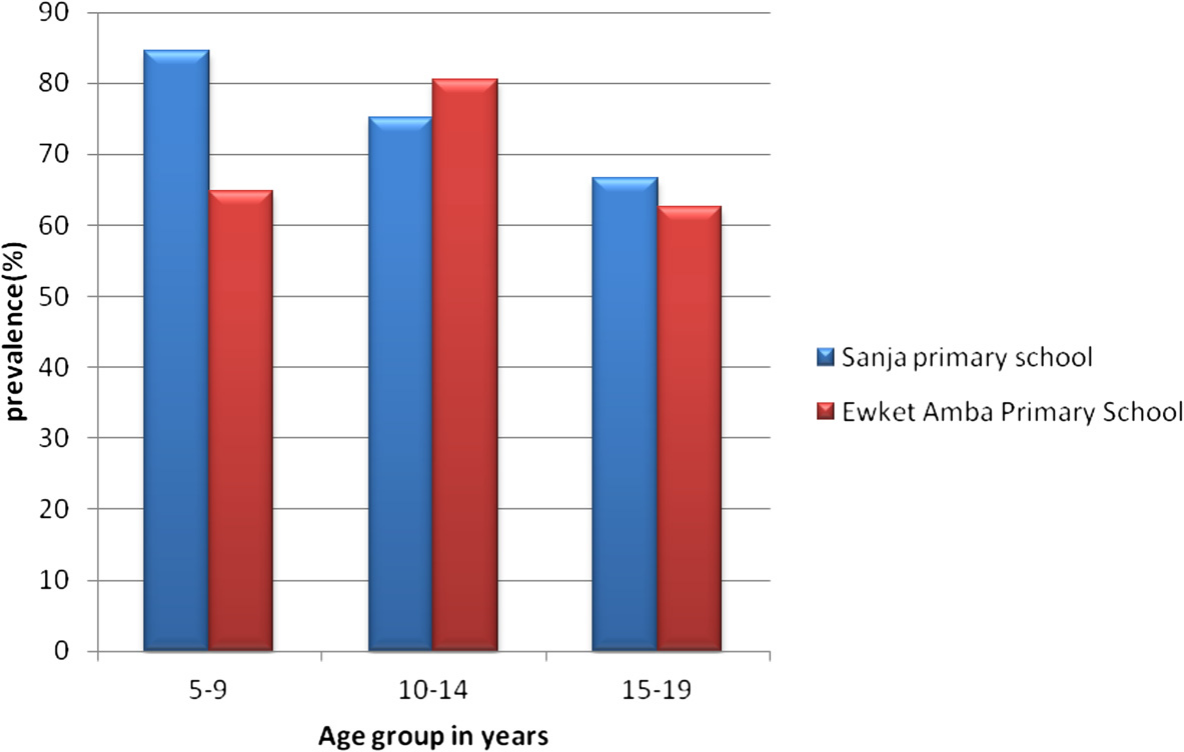
**Age groups distribution of S** ***. mansoni* infection by Kato-Katz method, 2013.**

**Figure 3:**
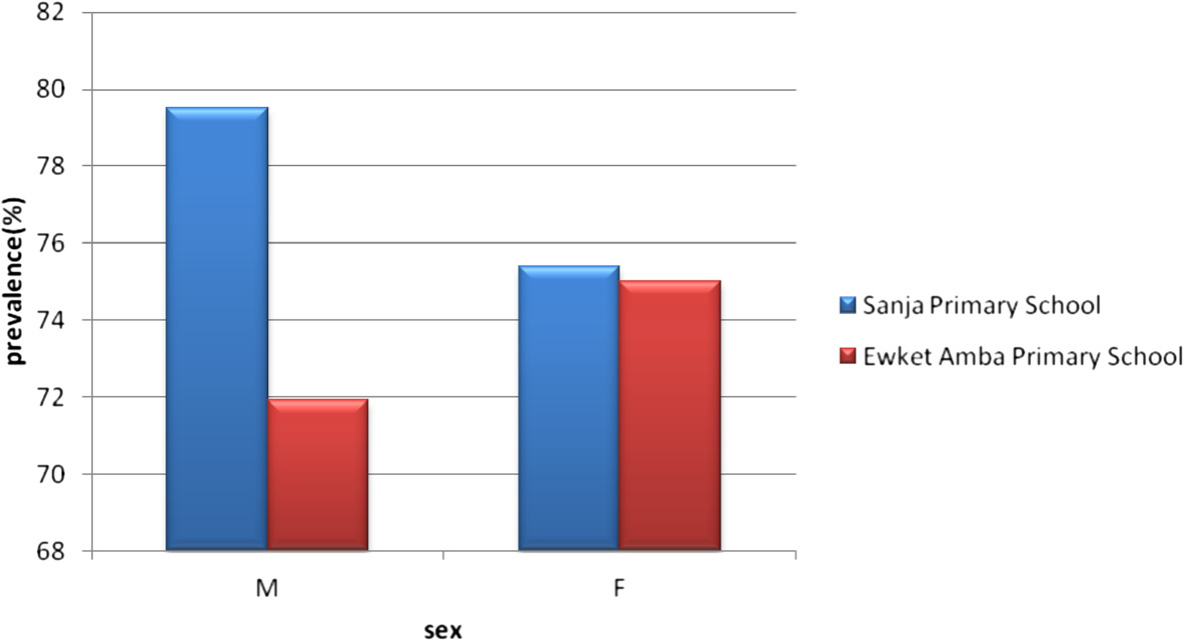
**Sex group distribution of S** ***. mansoni* infection by Kato-Katz method, 2013.**

As shown in Figure 2, the prevalence of *S. mansoni* infection was high (>60%) in all age groups of both schools. The highest infection rate (84.6%) was observed in children in the age group of 5–9 years followed by 75.2% in the age group 10–14 years of Sanja Primary school while in Ewket Amba Primary school 80.5% and 64.7% infection rate was observed in children at the age groups of 10–14 and 5–9 years, respectively. In both schools age had no statistically significant association with *S. mansoni* infection (P-values, 0.64 [Sanja primary school] and 0.23 [Ewket Amba primary schools]). The prevalence of infection using the Kato-Katz method among males and females was 79.5% and 75.4%, respectively in Sanja Primary school whereas 71.9% and 75%, respectively, in Ewket Amba primary school (Figure 3). In both schools sex also had no statistically significant association with *S. mansoni* infection (P-values, 0.44 and 0.68 for Sanja and Ewket Amba primary schools respectively).

**Figure 4:**
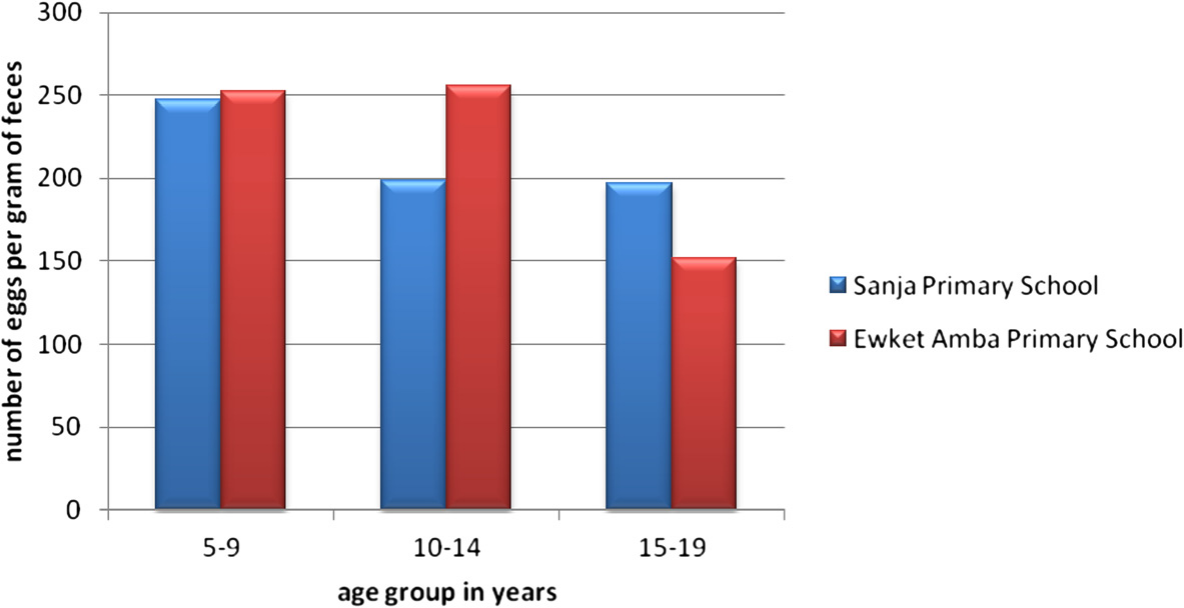
**Mean (GM) of *S. mansoni *** **egg count in relation to age groups, 2013.**

The geometric mean epg of *S. mansoni* infection was 211 and 251.8 in Sanja and Ewket Amba Primary schools, respectively. In Sanja Primary school the highest epg was observed among 5–9 years old children (247.6 epg) followed by 198.4 epg and 197.2 epg for the age groups 10–14 and 15–19 years, respectively while in Ewket Amba Primary school the highest epg was observed among 10–14 years old children (255.9 epg) followed by 251.9 epg and 151.8 epg for the age groups 5–9 and 15–19 years, respectively (Figure 4). In both schools age had no statistically significant association with intensity of *S. mansoni* infection (P-values, 0.97 and 0.70 for Sanja and Ewket Amba primary schools respectively). In Sanja Primary school the intensity of infection among male and female students was 205 epg and 217 epg, respectively, whereas intensity of infection among male and female students of Ewket Amba Primary school was 311.2 epg and 210.3 epg, respectively.

**Figure 5:**
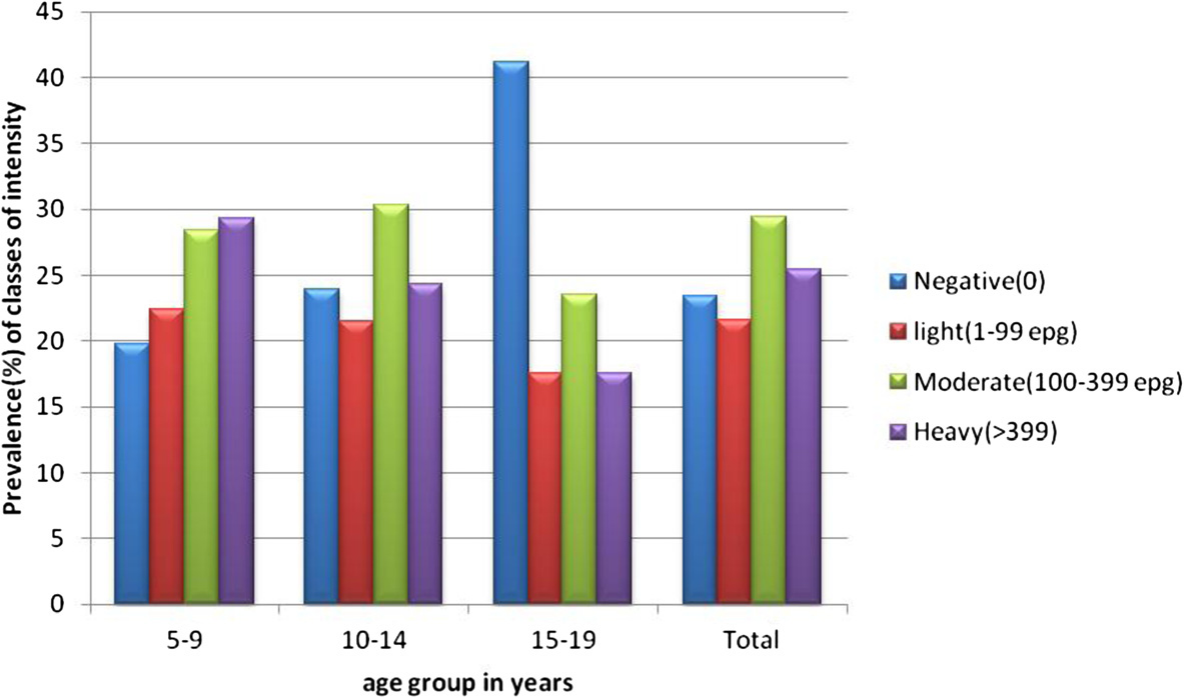
**Distribution of classes of intensity (GM) of *S. mansoni *** **infection among age groups.**

**Figure 6:**
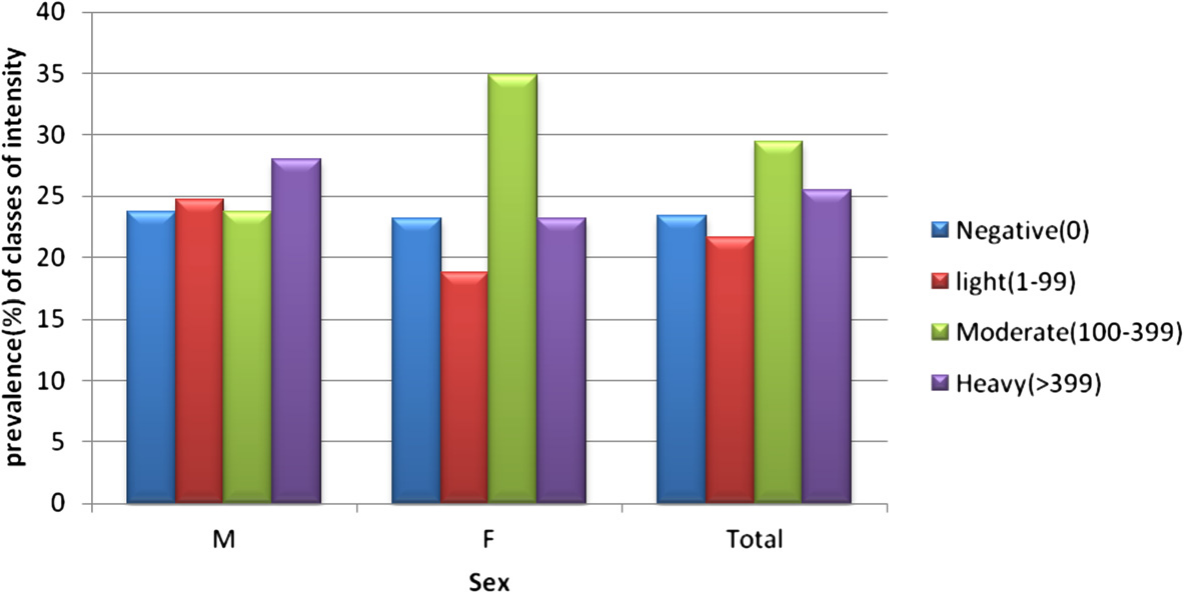
**Distribution of classes of intensity (GM) of *S. mansoni *** **infection among sex groups.**

As shown in Figures 5 and 6, the prevalence of light, moderate and heavy infections were 21.6%, 29.4% and 25.5%, respectively. The highest egg count was 6288 epg.

### Malacological survey

Sanja River and Maho stream were surveyed for *S. mansoni* intermediate hosts during January and April and snails were collected using a scoop. *Biomphalaria pfeifferi* were collected from both water bodies but very few *B. pfeifferi* were collected from Sanja River. *B. pfeifferi* were found attached to leaves falling from surrounding trees, stones, decaying wood, plastic, etc. Out of 154 *B. pfeifferi* collected during January, 89 were not observed to shed schistosome cercaria, 39 died during transportation and the remaining 26 (16.9%) shed schistosome cercariae. Out of 221 *B. pfeifferi* collected during April, 180 were not observed to shed schistosome cercaria, 35 died during transportation and the remaining 6 (0.027%) shed schistosome cercaria. The size of infected and non-infected *B. pfeifferi* in both periods was different. During January, the sizes of infected and non infected snails were between 7.8 mm-12.6 mm and 5.2 mm-11.9 mm respectively. The sizes of infected and non infected snails in April were between 7 mm-11.8 mm and 5.1 mm-7.8 mm respectively.

Other snail species such as *Bulinus forskali* and *Lymnaea natalensis* were also collected from Maho stream and Sanja River but they did not shed cercaria.

Physical characteristics of the water bodies during the snail survey showed that Maho stream was moderately turbid, and covered by large amounts of weeds, algae and other garbage such as plastic, clothes and fallen leaves. It was small and slow flowing. The substratum of the water body was muddy and sandy. On the contrary, Sanja River was large, fast flowing, clear and lacked any vegetation, weeds and algae. The substratum of the water was sandy.

*S. mansoni* infection was successfully established in laboratory mice and 209 male, 49 female and 113 in copula adult worms were harvested after six weeks of laboratory maintenance.

### Risk factors for *S. Mansoni* infection

Direct observations showed that people excreted in open fields, particularly in groves and shades of trees near to both water bodies. The inhabitants also bathed and washed their clothes in the river. The result from structured questionnaires had shown that swimming, washing clothes, and bathing in the river were associated with high risk of *S. mansoni* infection (Table 2).

**Table 2:** **Association of *S. mansoni *** **infection with river water contact habit and demographic factors of the study participants, 2013**

**Risk factors**		***S. mansoni***		**OR**	**95.0% C.I. for OR**		**P-value**
		**Positive**	**Negative**		**Lower**	**Upper**	
Swimming	Yes	261	65	3.26	1.819	5.853	0.00
	No	32	26				
River contact while crossing	Yes	250	74	1.34	0.72	2.479	0.359
	No	43	17				
Washing clothes in the river	Yes	259	62	3.56	2.02	6.286	0.00
	No	34	29				
Bathing in the river	Yes	244	62	2.38	1.39	4.08	0.002
	No	48	29				
Fetching water from river	Yes	179	50	'1.29	0.801	2.071	0.297
	No	114	41				
Sex	M	143	44	1.02	0.64	1.63	0.94
	F	150	47				
Age	5-9	89	27	1.14	0.73	1.78	0.58
	10-14	193	58				
	15-19	11	6				

Children who swam in river had 3.26 times higher odds of being infected with *S. mansoni* than those who did not have a swimming habit. Children, who wash clothes and bathe in the river, had 3.56 and 2.38 times higher odds of being infected with *S. mansoni* than who did not, respectively (Table 2).

## Discussion

New schistosomiasis mansoni transmission foci are being discovered in different parts of Ethiopia [21]. For establishment of schistosomiasis in new transmission foci, the ecologies of the schistosomes, appropriate aquatic snail intermediate hosts and the human definitive host must converge in space and time in suitable water bodies. In the present study, the observation that children were excreting *S. mansoni* ova, the shedding of schistosome cercariae by *B. pfeifferi* collected from the two water bodies in Sanja town, and the establishment of *S. mansoni* life cycle in laboratory bred mice are confirmations of the endemicity of *S. mansoni* in Sanja Town. This endemic focus might have existed for an undetermined period of time or may be a newly established one as a result of population movement into the region from other endemic localities or infected snail intermediate host transfer into the water bodies [16].

The prevalence of schistosomiasis mansoni observed in the present study was very high compared to most studies conducted among school children in different parts of Ethiopia which reported prevalence ranging from 30 to 70% [22-27]. The high prevalence of *S. mansoni* in the present study may be attributable to indiscriminate defecation and inadequate provision of potable water. In the present study area, piped water was inadequate to meet the demands of the residents and Sanja River and Maho stream serve as sources of water for laundering, bathing and other domestic and recreational purposes. Most of the town dwellers had no toilet and deposited their feces in open fields, particularly near the water bodies.

Site specific prevalence of *S. mansoni* infection was slightly higher among children in Sanja Primary School (77.4%) than Ewket Amba Primary School (73.5%) although this difference was not statistically significant (p > 0.05). The reason behind this difference could be the proximity of Sanja primary school to both water bodies and Ewket Amba Primary School was far away from Maho stream from which large number of *B. pfeifferi* were collected.

Previous studies have shown that males are the more affected by schistosomiasis than females [25,28-30]. This was explained by the fact that males had higher frequency of contact with cercariae infested water bodies than females. However, in the present study both females and males were equally affected indicating similarities in water contact behavior of the male and female children in the study area.

As an indication that children aged 5–9 years are equally active as the 10–14 years of age group in their water contact behavior, both age groups were equally infected with *S. mansoni*. The similarity in infection rate was substantiated by the similarity in the age specific intensity curve of *S. mansoni* infection in both age groups in Sanja and Ewket Amba Primary schools indicating an intensive exposure of both age groups from both schools in the two infected snail infested water bodies. This is because both school children may make contact with the water bodies after school as the town is too small. This suggests a unique epidemiology of schistosomiasis in the area contrary to the reports from other parts of the country where the age group 10–14 have higher infection prevalence than the 5–9 year olds [29,31-34]. The fact that the mean intensity of *S. mansoni* among Sanja and Ewket Amba Primary schools was much higher from other previously established foci in the country could be an indication of lack of availability of drug treatment in Sanja Town, which is a locality not listed as a schistosomiasis endemic area until the present study.

Contrary to several reports from other parts of the country [35-37] the prevalence of non-schistosome intestinal parasites among school children in Sanja Town was very low and the community appears to represent a low risk population in this regard. Among other factors, such variations are attributable to differences in environmental conditions [38]. That is, the combination of high temperature and long dry season in the study area, which is known to be not favorable for the transmission of other intestinal parasites [39].

The snail survey showed more abundance of *B. pfeifferi* in Maho stream and relatively few numbers in Sanja River. The reason for the observed difference in abundance of *B. pfeifferi* in the two water bodies could be explained by the fact that Maho stream is slow flowing and is abundantly covered with aquatic weeds compared to Sanja River. It has been shown that small rivers with flow rate of 10–30 cm/second, with slight turbidity, abundant vegetation at the edge and muddy, are potentially favorable habitats for *B. pfeifferi* and other snails including *Bu. forskali*[40].

Measurement of infection rates by monitoring the number of infected local snails in field snail populations is one of the basic tools for studies on the epidemiology of schistosomiasis to estimate the transmission potential of an area [41]. In the present study high infection rate of snail intermediate host was observed and this indicates that there is compatibility between the snails and the *S. mansoni* parasite. This also implies that there is high transmission potential of *S. mansoni* infection in the study area. In the present study, the reason for infection rate variation in the snail population could be lack of exposure to *S. mansoni* miracidia and the reason for the variation of size and density of snails in the two sampling periods could be the growth of snails inhibited by their density. Though not studied systematically, another possibility for this variation could be mortality of snails during exposure to *S. mansoni* that could decrease the density of snails [42].

Analysis of water contact habits of the study population confirmed that swimming, washing clothes and bathing in the river was significantly associated with high risk of *S. mansoni* infection. The results are in agreement with the observations of studies conducted in Kenya and Malawi [33,43]. The main reason for this association could be the proximity of the schools to the water bodies infested with infected snails.

## Conclusion

The finding of *S. mansoni* infected young children, the collection of *B. pfeifferi* infected with schistosome cercariae, and the establishment of infection in lab-bred mice all confirmed the transmission of schistosomiasis mansoni in Sanja area. The prevalence and intensity of schistosomiasis mansoni among school children was high and the area represents high risk community. Human contaminative activities such as open field defecation and exposure activities such as washing, swimming and bathing in the two water bodies on the area favors transmission of schistosomiasis in Sanja Town. Therefore, based on the finding of the study, we recommend that there should be mass drug administration with Praziquantel; treating water bodies with available molluscicides to reduce the transmission of the disease; provision of safe water supply and sanitary facilities; continuous health education on the means of transmission of *S. mansoni* infection and its control and constructing bridges on the two water bodies to reduce water contact while crossing the rivers.

## Competing interests

The authors declare that they have no competing interests.

## Authors’ contributions

GA is principal investigator; MA identified the study area; BE and BP worked as advisors. All authors read and approved the final version of the manuscript.
